# Current status and future application of electrically controlled micro/nanorobots in biomedicine

**DOI:** 10.3389/fbioe.2024.1353660

**Published:** 2024-01-19

**Authors:** Ruochen Pu, Xiyu Yang, Haoran Mu, Zhonghua Xu, Jin He

**Affiliations:** ^1^ Jintan Hospital Affiliated to Jiangsu University, Changzhou, Jiangsu Province, China; ^2^ Shanghai Bone Tumor Institution, Shanghai, China; ^3^ Department of Orthopedics, Shanghai General Hospital, Shanghai Jiao Tong University School of Medicine, Shanghai, China

**Keywords:** electrically-controlled MNRs, MNRs, application, fabrication, Janus 1

## Abstract

Using micro/nanorobots (MNRs) for targeted therapy within the human body is an emerging research direction in biomedical science. These nanoscale to microscale miniature robots possess specificity and precision that are lacking in most traditional treatment modalities. Currently, research on electrically controlled micro/nanorobots is still in its early stages, with researchers primarily focusing on the fabrication and manipulation of these robots to meet complex clinical demands. This review aims to compare the fabrication, powering, and locomotion of various electrically controlled micro/nanorobots, and explore their advantages, disadvantages, and potential applications.

## 1 Introduction

Micro/nanomotors (MNRs), characterized by their small size and precise control capabilities, represent a class of intelligent robots operating in the micro- and nanoscale dimensions. These robots possess the ability to navigate freely within the microscopic world and harness external environmental energy for propulsion and manipulation (Feng et al., 2023). As a result, MNRs have found extensive applications in the field of modern medicine, including targeted drug delivery, precision healthcare, biosensing, and waste removal (Vujacic Nikezic and Grbovic Novakovic, 2022; Zhou et al., 2022). Current research endeavors predominantly focus on magnetic (Diller et al., 2013; Peyer et al., 2013; Servant et al., 2015; Li et al., 2019; Xie et al., 2019; Fang et al., 2023), optical (Wang J. et al., 2019; Dai et al., 2022; Wang et al., 2023), acoustic (Abbas et al., 2020; Das et al., 2022; Wu et al., 2023), thermal (Tu et al., 2017), and biohybrid MNRs (Park et al., 2013; Han et al., 2016; Wang X. et al., 2019; Magdanz et al., 2021; Song et al., 2022). In contrast, studies pertaining to electro-controlled MNRs remain limited, considering the intricate balance between fabrication complexity, cost-effectiveness, feasibility of energy supply, and existing clinical demands. From a technological standpoint, the materials employed in electro-controlled MNRs are often constrained by the limitations imposed by their motion mechanisms, necessitating a certain level of conductivity or charge-carrying capability. Consequently, the development of novel materials for electro-controlled MNRs is hindered (Demirörs et al., 2018). In terms of energy supply, the storage and transmission of energy at the micro- and nanoscale are inherently limited, thus rendering electro-controlled MNRs reliant on external power sources or wireless energy transfer for their energy needs. This complexity and dependence on external sources impose significant constraints on the feasibility of electro-controlled MNRs in practical applications (Fath et al., 2022).

**FIGURE 1:**
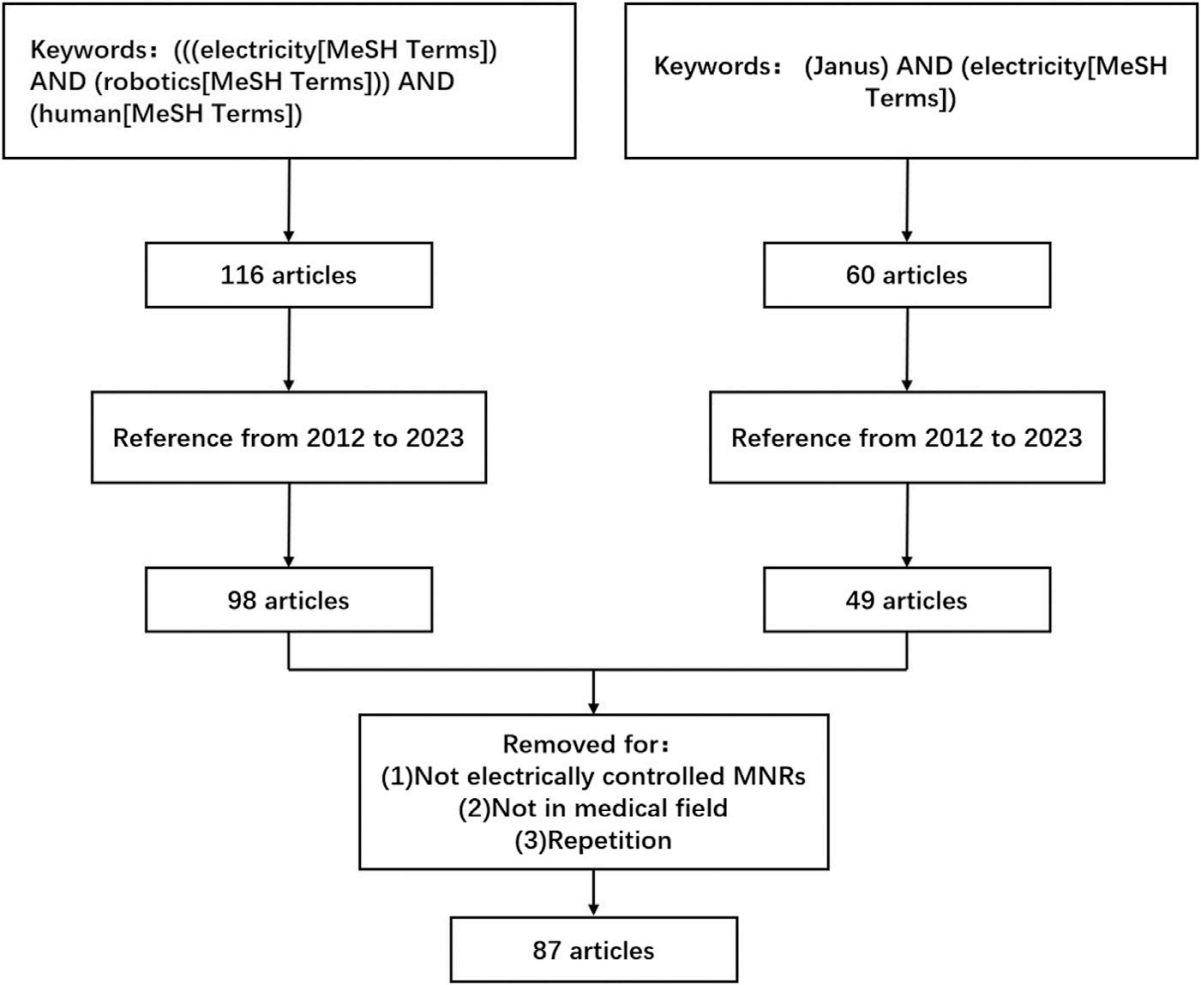
A Review strategy.

However, compared to other MNRs, electro-controlled MNRs still possess several advantages. Firstly, electric fields offer high energy efficiency and a wide range of modulation, enabling efficient propulsion of MNRs at extremely low voltages while exhibiting different motion capabilities and patterns at various voltage levels (Kim et al., 2017; Shen et al., 2020; Lee et al., 2021). Secondly, electro-controlled MNRs can alter their shape and mechanical properties through the adjustment of electric fields, allowing adaptation to diverse application requirements (Jang et al., 2021; Liu et al., 2021; Zheng et al., 2022). Moreover, in drug delivery, electro-controlled MNRs offer unique advantages. As carriers, they not only achieve precise motion but also enable precise drug release through external electric fields, facilitating ideal drug administration. This review summarizes the fabrication, energy supply, and motion mechanisms of electro-controlled MNRs, compares their relative advantages, and emphasizes the need for further research in terms of biocompatibility and propulsion. By evaluating existing studies, the review provides insights into the future clinical applications of electro-controlled MNRs (Figure 1).

## 2 Fabrication of electro-controlled MNRS

The primary challenge in the fabrication of micro-motors lies in achieving robust locomotion of robots in low Reynolds number fluids, such as blood, at the nanoscale (Zheng L. et al., 2023). In such fluids, inertial forces are exceedingly small, while MNRs often require precise control, necessitating continuous power input (Wang, 2009; Soto et al., 2020). Furthermore, conventional fuels like chemical propellants are typically biologically harmful (Lv et al., 2023), prompting biomedical researchers to demand more compatible propulsion methods. In addition to these considerations, researchers must also address cost and fabrication complexity to meet the growing practical demands of clinical applications.

During the fabrication process of MNRs, several factors significantly influence the design. i) Energy sources for MNRs: These robots can be broadly categorized into externally propelled and self-propelled types. For externally propelled MNRs, precise control of the external field on the robot particles is crucial, encompassing both collective and individual configurations (Zheng L. et al., 2023). The external field can also serve as a means of recharging the robots, for instance, through wireless charging techniques (Kim et al., 2017). Conversely, self-propelled MNRs often rely on chemical and biological energy sources, with common catalytic media including light (Dong et al., 2018; He et al., 2021), electricity (Seh et al., 2017; Suen et al., 2017), and chemical environments (Lyu et al., 2021). Consequently, the fabrication process must consider not only the MNRs themselves but also the design of their accompanying external fields. ii) Modes of motion for MNRs: In addition to conventional field-guided motion, MNRs can exhibit a diverse range of motion patterns based on their design. Under external field stimulation, these robots can achieve rotation, translation, and helical propulsion, among other modes (Lee et al., 2023). For electro-controlled self-propelled MNRs, specific motion behaviors can be achieved through the design of structures such as Janus particles, which exploit distinct hemisphere configurations (Shen et al., 2020). Additionally, biomimetic structures, including flagella-like structures (Ma et al., 2012), sperm-like structures (Celi et al., 2021; Mayorga-Martinez et al., 2021), and cilia-like structures (Wang W. et al., 2022), offer the ability to facilitate flexible forward and backward motion and directional changes. In the context of electro-controlled MNRs, careful consideration must also be given to the manipulation methods, such as the selection between alternating and direct current fields, as well as the design of microfluidic chips.

Currently, common methods for the fabrication of MNRs include Photolithography (Iacovacci et al., 2019; Mu et al., 2021), Template-guided electrodeposition (Ji et al., 2021; Li et al., 2021; Lu et al., 2022), Additive manufacturing (Otuka et al., 2014; Li and Pumera, 2021; Liu et al., 2022), and Glancing angle deposition (Cohen and Walt, 2019; Ghosh and Ghosh, 2020; Dasgupta et al., 2022). Photolithography involves the use of photosensitive materials and a photomask. The light emitted by a photolithography machine passes through the patterned mask and exposes a thin film coated with photoresist. The photoresist undergoes a change in properties upon exposure, allowing the pattern from the mask to be replicated onto the film, resulting in the formation of corresponding protruding structures (del Barrio and Sánchez-Somolinos, 2019). Template-guided electrodeposition utilizes electric current to reduce metal ions (or conductive polymers) onto the surface of an electrode covered with a porous membrane (Medina-Sánchez et al., 2018). In this process, adjusting the pore size of the membrane enables control over the size of the robot, and the porous membrane can be dissolved after fabrication to release the robot. Currently, there are five main additive manufacturing (also known as 3D printing) techniques used for the fabrication of MNRs: Direct laser writing (DLW), Stereolithography, Microscale continuous optical printing, Inkjet printing, and PolyJet (Zheng L. et al., 2023). Glancing angle deposition is a novel thin film deposition technique that involves controlling the tilt and rotation of the substrate to obtain nanostructured porous films different from traditional dense films. With the assistance of a rotating substrate, it is possible to achieve nanopores with various diameters and shapes (Wang Z. et al., 2022).

**FIGURE 2:**
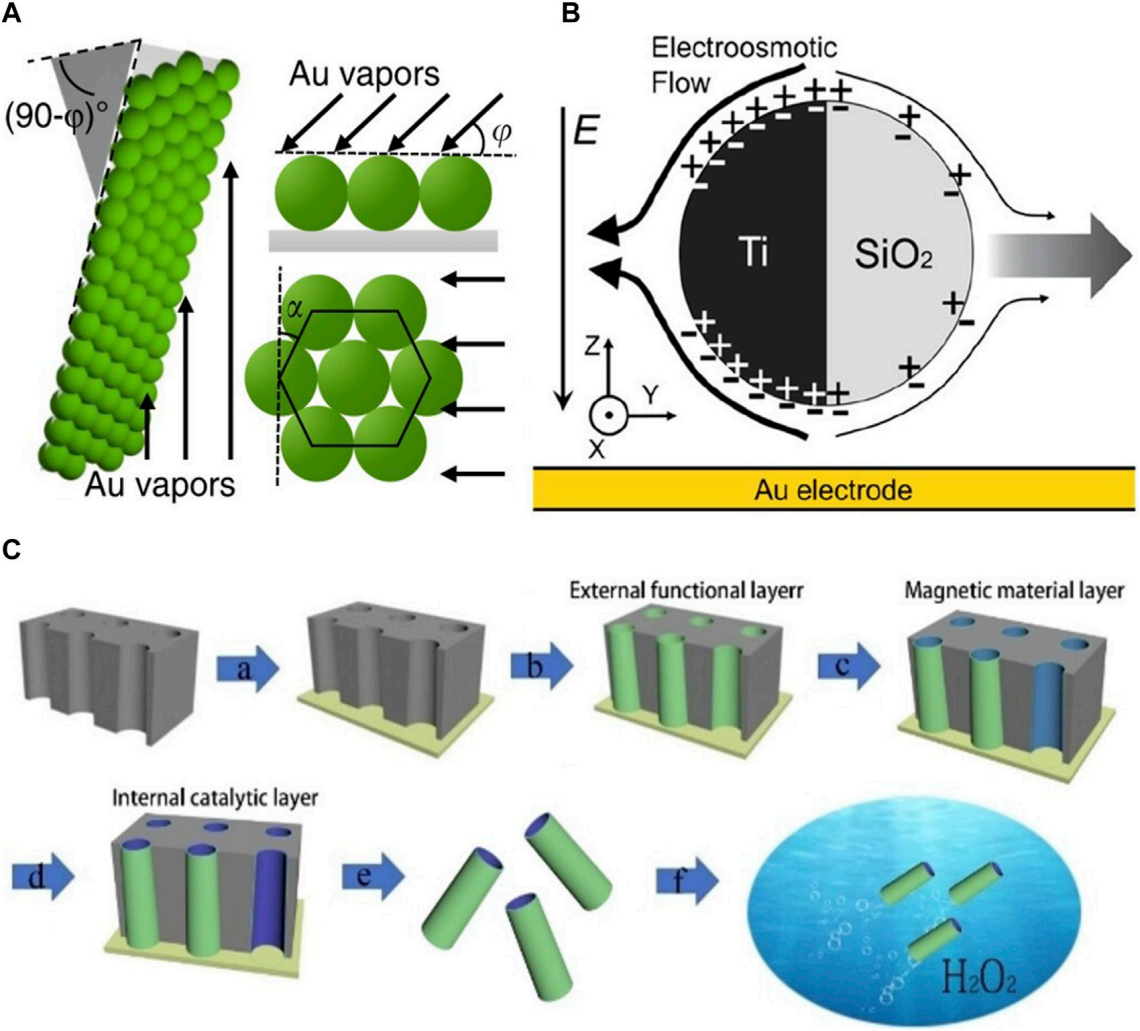
**(A)** A schematic of metal (Au) deposition using glancing angle deposition onto a substrate coated with a monolayer of polystyrene (PS) microspheres. The arrows indicate the direction of incident metal vapors (Lee et al., 2019). 2019, The Author (s). **(B)** Schematic of a SiO_2_–Ti particle undergoing induced charge electrophoresis (IECP) (Zhang et al., 2019). 2019 American Chemical Society. **(C)** Schematic diagram of the steps involved in the preparation of microtubules by template-assisted electrodeposition (Li et al., 2021). 2021 The Authors. Published by Wiley-VCH GmbH.

**FIGURE 3:**
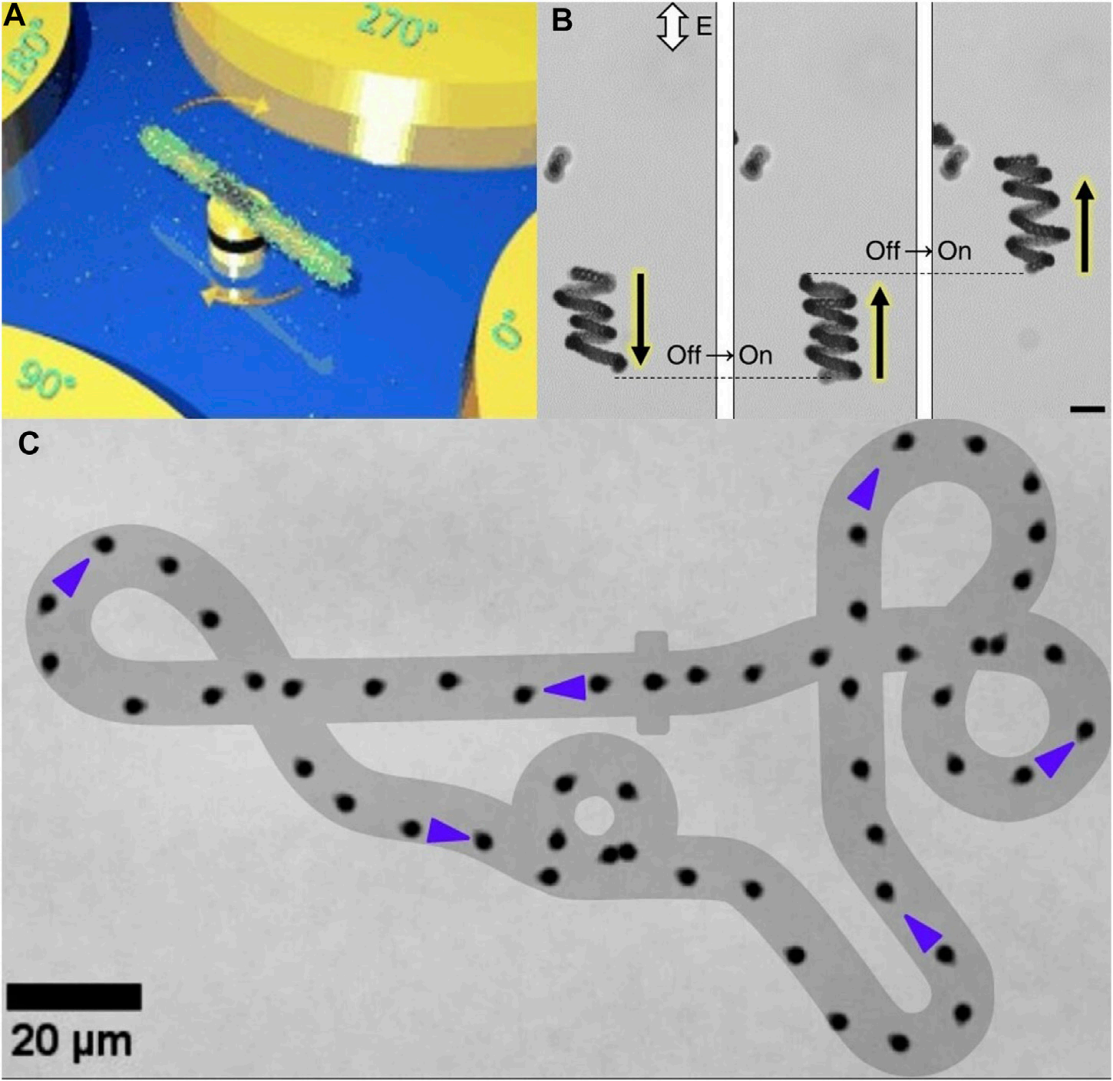
**(A)** Nanomotors accomplish rotation and drug release due to polarisation in the presence of electric tweezers (Xu et al., 2015). 2015 WILEY-VCH Verlag GmbH and Co. KGaA, Weinheim **(B)** Under an alternating electric field, particles exhibit helical motion and undergo chiral turnover (Lee et al., 2019). 2019, The Author (s) **(C)** Under concentration polarisation electrophoresis (CPEP), Janus particles move along predetermined orbits (Katzmeier and Simmel, 2023). 2023, The Author (s).

The fabrication of electrically controlled MNRs primarily focuses on the preparation of Janus particles. Given material constraints, common techniques for fabricating electrically controlled Janus particles include electron beam deposition (Demirörs et al., 2018; Zhang et al., 2019). Demirörs et al. Demirörs et al. (2018) used 4.64 μm diameter silica particles to prepare sub-monolayer structures. They coated a suspension of these particles onto a glass substrate and formed a sub-monolayer after drying. Subsequently, they employed the Plassys II (Plassys Bestek) electron beam deposition technique to vertically deposit a 15 nm thick layer of platinum onto the sub-monolayer. The researchers then subjected the samples to ultrasonication in deionized water to collect the particles, which were subsequently collected by centrifugal concentration. Similarly, by depositing a 15 nm nickel layer and a 15 nm platinum layer onto a dried colloidal sub-monolayer, Janus particles with magnetic responsiveness can be prepared. Similarly, Zhang et al. Zhang et al. (2019) used drop casting to prepare 3 nm single-layer SiO2, which was then suspended in 20 μL of ethanol and dispersed by ultrasonication (Figure 2B). After drying, a 50 nm titanium film was deposited onto the silica microspheres using electron beam deposition, followed by ultrasonic collection. Building upon these fabrication methods, Lee et al. (2019) employed glancing angle deposition to deposit size- and shape-tunable 10 nm chromium and 30 nm gold onto polystyrene microspheres at small angles of 0° < φ < 20° (Figures 2A, 3B). These nearly symmetric mirror-like triangular structures endowed the particles with unique motion capabilities and trajectories.

For electrically controlled MNRs in forms other than Janus particles, electron beam deposition and photolithography techniques are also commonly used for their fabrication. Fennimore et al. Fennimore et al. (2003), for example, used photolithography to attach multiwalled carbon nanotubes with 10 nm chromium and 90 nm gold, creating rotatable nanoscale motors.

Overall, the fabrication of electro-controlled MNRS does not differ significantly from other types of micro- and nanomotors. Rather, the fabrication of electro-controlled MNRS tends to be relatively limited due to the constraints of material properties (Figure 2C). MNRs driven by electric fields require materials with good conductivity, while electrically controlled MNRs based on Janus particles require specific electrically responsive materials. However, research on conventional polymers and metals for these applications is relatively mature. Currently, research on the fabrication of electro-controlled MNRS should focus on two directions: i) Optimizing existing fabrication methods and material performance to achieve the production of conventional robots through lower cost and lower complexity approaches. ii) Integrating MNRs with biological structures to create artificial muscles, bio-hybrid electrically responsive robots, and so on, and conducting in-depth research on biocompatibility and autonomous locomotion.

## 3 Energy acquisition of electro-controlled MNRS

The energy for electro-controlled MNRS primarily originates from external electric fields and self-propulsion (Wang and Zhang, 2021). Electro-controlled MNRS powered by external electric fields can be categorized into two types: i) Direct external field-driven motors (Kim et al., 2017; Lee et al., 2019; Zhang et al., 2019; Shen et al., 2020; Demirörs et al., 2021; Zheng Y. et al., 2023; Haque et al., 2023). This method of energy supply is the most prevalent and offers advantages such as simplicity, contactless operation, and minimal waste generation. ii) External field-powered motors (Guanying et al., 2007; Lv et al., 2022). This non-contact power supply mode avoids the need for intricate wiring connections and finds promising applications in human environments. Once charged, these micro- and nanomotors acquire the ability for autonomous motion within a certain time frame, enabling them to perform more precise movements in complex environments. Self-propelled electro-controlled MNRS primarily exist in the form of electrochemical Janus particles (Nie et al., 2018; Lee et al., 2021; Liu et al., 2021; Yang et al., 2022; Liao et al., 2023), which will be discussed further in subsequent sections.

### 3.1 Electrically controlled MNRs operated by direct external fields

To respond to external electric fields, electric field-driven MNRs require materials that possess inherent conductivity. Two types of electric fields, alternating current (AC) and direct current (DC) can be employed for remote control of MNRs’ propulsion. Common propulsion mechanisms include electrostatic tweezers (Fan et al., 2011), induced charge electrophoresis (ICEP) (Gangwal et al., 2008), electrophoretic flow (Calvo-Marzal et al., 2010), electrohydrodynamic flow (EHD) (Ma et al., 2015), and self-electrophoresis (sDEP) (Boymelgreen et al., 2016).

#### 3.1.1 MNRs manipulated by electrostatic tweezers

Electrostatic tweezers are a technique that employs an alternating electric field applied to patterned electrodes to manipulate suspended nanoparticles. This technique allows for controlled rotation, positioning, and cargo release of nanowires, among other functionalities (Fan et al., 2011). Xu et al. Xu et al. (2015) demonstrated the polarization effect of electrostatic tweezers on a specific type of micro- and nanorobot composed of plasma-sensitive nanorods as rotors, patterned nanomagnets as bearings, and microelectrodes as stators (Figure 3A). They systematically validated the polarization effect of electrostatic tweezers on this type of micro- and nanorobot, which can be utilized to modulate the release rate of biochemical substances on the surface of nanoparticles, demonstrating its potential for drug loading and release applications. Furthermore, the alternating magnetic field required for electrostatic tweezers can also be generated under optical induction. Zhang et al. (2022) provided a detailed overview of the working mechanism and experimental setup of optoelectronic tweezers, along with its applications in non-biological and biological fields, indicating the future directions of electrostatic tweezers.

#### 3.1.2 MNRs manipulated by electrophoretic flow

Another method for manipulating MNRs using an AC electric field is electrophoretic flow. Electrophoretic flow is the fluid motion induced by applying a voltage across porous media, microchannels, and other fluidic conduits, relying on the generated dipole moment (Alizadeh et al., 2021). The advantages of electrophoretic driving include a simple system architecture, ease of operation, and a planar flow profile. However, electrophoretic driving is susceptible to factors such as applied electric field strength, channel surface, microfluidic properties, and heat transfer efficiency, resulting in relatively poor stability. Additionally, this driving mechanism is only applicable to electrolyte solutions, limiting its range of applications. Calvo-Marzal et al. (2010) utilized an AC electric field to drive PPy-Cd/CdSe-Au-CdSe hybrid fuel-free semiconductor diode nanowires based on the electrophoretic flow mechanism, enabling motion along the direction parallel to the electric field axis. This structure can easily attach drugs, biosensing structures, and other entities, making it promising for biomedical applications.

#### 3.1.3 MNRs manipulated by electrohydrodynamic flow

By applying a high voltage between two electrodes, an electric field is formed in an electrolyte solution. When the applied voltage exceeds a certain threshold, corona discharge occurs, causing ions of the same polarity as the electrode to move towards the other electrode, creating space charges. The resulting current induces Coulombic forces between the ions, leading to electrohydrodynamic flow (Peng et al., 2023). Ni et al. (2017) employed sequential capillary-assisted particle assembly (sCAPA) to connect microspheres of different materials into defined-shaped mixed clusters, referred to as “colloidal molecules.” These clusters can actively translate, circulate, and rotate under the driving force of asymmetric electrohydrodynamic flow. Clustered particles possess motion characteristics and cargo-loading capabilities that individual nanoparticles lack, making them advantageous for targeted drug delivery.

Comparing electrophoretic flow and electrohydrodynamic flow, both involve electrokinetic phenomena that induce fluid motion through an applied electric potential. Electrophoretic flow offers high efficiency, controllability, low diffusion, and simple devices but has relatively low flow velocities, sensitivity to interfering charges, and complex control requirements (Slater et al., 2010). On the other hand, electrohydrodynamic flow can enhance mixing at the microscale and has the potential for electrochemical-driven self-propulsion, but its research is still in its early stages, and control techniques are lacking. Further research is needed to address the limitations and explore the full potential of electrohydrodynamic flow.

#### 3.1.4 MNRs manipulated by induced charge electrophoresis

Induced charge electrophoresis (ICEP) is a special nonlinear electrokinetic phenomenon (Gangwal et al., 2008). Generally, in a non-uniform alternating current field, net electrostatic forces induce particle motion, a phenomenon known as dielectrophoresis. Building upon this, Murtsovkin, (1996) discovered that the polarization of the ion double layer can also cause nonlinear electro-osmotic flow at low frequencies (kHz). Based on this, Bazant and Squires, (2004) introduced the term induced charge electrophoresis to describe the collective motion resulting from the action of an externally applied electric field on the self-induced diffusive charges near a polarizable surface.

Unlike electrophoretic flow, ICEP is a driving method commonly used for Janus particles. The direction of particle motion is perpendicular to the field direction. This is because, in an electric field, the metal hemisphere of a Janus particle polarizes more strongly than the dielectric hemisphere, resulting in a higher electro-osmotic strength on the metal side. Lee et al. Lee et al. (2023) applied an external alternating electric field to a cluster of Janus particles and demonstrated the diverse motion modes generated by ICEP, showcasing its rich potential.

This manipulation method of MNRs distinguishes itself from electrophoretic flow and electrohydrodynamic flow by directly acting on the particles themselves, with the particle motion direction typically different from the electric field lines (Figure 3C). Compared to traditional electrophoresis, this electrophoresis mode has more limited applications but provides higher precision, making it highly valuable in the field of electrically controlled microactuators.

### 3.2 MNRs manipulated by electrohydrodynamic flow

With the increasing demand for painless diagnosis, high security, and accurate detection, wirelessly powered MNRs have become a hot research area (Gao C. et al., 2021). Guanying et al. (2007) proposed an inductively coupled wireless power transfer system employing two coils. The coupling coefficient between these coils was measured based on axial, transversal, and spacing deviations. By selecting suitable tuning capacitors and transmission frequencies, the researchers derived and optimized the power transfer efficiency. Compared to the parallel resonant circuit topology, the series resonant circuit (SRC) demonstrated greater adaptability on the receiving end. To address the uncertainty in the direction of the receiving coil, the researchers also proposed and studied a multi-coil receiving structure. Experimental results showed that when the receiving coil was positioned at the center of the transmitting coil, the system could stably receive up to 170 mW of direct current power with an efficiency of 1.3%, meeting the power requirements of certain microsystems. Although researchers have made significant progress in miniaturizing the structure, the design and fabrication of nanoscale wirelessly powered robots remain relatively unexplored territory.

### 3.3 MNRs manipulated by other methods

In addition to external field powering, converting other forms of energy into electricity is also a common method of energy supply for MNRs. Fan et al. introduced the concept of Triboelectric Nanogenerators (TENG) in 2012, which can directly convert mechanical energy from our daily activities into electricity and has found extensive applications in engineering (Zhong et al., 2013; Wang et al., 2017). TENG exhibits high output voltage and low output current characteristics. The short-circuit current is typically in the milliampere or microampere range, while the open-circuit voltage can reach several kilovolts (Lee et al., 2015; Seung et al., 2015). Lv et al. (2022) designed a TENG that can be driven by ultra-weak mechanical stimuli. The study found that by adjusting the driving frequency, separation distance, and motion amplitude, a maximum energy conversion efficiency of 73.6% and an energy output of 48 pJ can be achieved. This work holds significant importance in expanding the applications of TENG in the field of biomicrorobotics. Furthermore, Nie et al. (2018) combined TENG with electrowetting techniques to achieve self-powered manipulation of microfluidics. They developed a miniature car with four droplets as wheels, limiting its volume to tens of nanoliters, and accomplished precise stepping motion and cargo loading.

Moreover, the idea of converting mechanical energy into electricity can potentially be integrated with other micro/nanorobot designs. Lee et al. (2021) designed a nanobiological supercapacitor (nBSC) catalyzed by redox reactions, which has a volume of only 1 nL and operates at high potentials ranging from 1V to 1.6V. To withstand the pressure and temperature variations in blood flow, the research team utilized a planar structure that self-assembles into a compact three-dimensional tubular geometry. Combining this tubular structure with energy conversion systems like TENG may potentially enable the development of MNRs with high energy storage capacity and continuous motion/supply.

Bubble propulsion is an important mode of propulsion in liquid environments. In electrochemistry, there are various methods to generate a large number of bubbles, which provides an exciting theoretical basis for the integration of electrically controlled micro/nanorobots with bubble propulsion. Zhan et al. (2023) have developed a photopyroelectric slippery surface (PESS) based on the photopolymerization-electric effect to achieve versatile manipulation of bubbles. Under near-infrared light irradiation, the PESS generates dielectric wetting and nonuniform electric fields, resulting in the simultaneous application of the Laplace force and dielectrophoresis force on the bubbles, enabling high-speed movement. These bubbles can be efficiently and precisely guided along arbitrarily designed paths, covering a wide range of volumes. More importantly, PESS allows for the splitting, merging, and detachment of underwater bubbles, offering a promising approach for selective chemical reactions, self-assembly, and cargo transportation.

## 4 Motion modes of electrically controlled MNRs

As mentioned earlier, electrically controlled MNRs generally move under the influence of electric fields or electric field-induced fluid flow. This mode of motion typically relies on the direction and strength of the electric field, making the motion of MNRs predictable and highly controllable (Yang et al., 2020; Wang et al., 2021). In addition to linear and curved motion, common motion modes include rotation, vibration, and spiral movement. Individual MNRs often exhibit limited motion states, such as linear motion or controllable motion under orbital confinement. Brooks et al. achieved the spiral motion of spherical colloids with low-symmetry metal patches in an alternating current electric field (Lee et al., 2019). However, when MNRs move collectively, they can demonstrate a wider range of motion patterns. Lee et al. (2023) utilized asymmetric Janus particle clusters to achieve five different states of motion: translation, rotation, butterfly pattern, spiral, and orbital motion. This enables electrically controlled MNRs to have enhanced motion capabilities in complex three-dimensional environments.

In different modes of motion, electrochemically-controlled micro/nano robots can adapt to different environments to demonstrate multitasking capabilities. Here are some basic motion modes and their application: i) Rolling: Electrically controlled MNRs that can roll have potential applications in targeted drug delivery within the bloodstream. Their rolling motion enables them to navigate through blood vessels, reaching specific target sites for drug release. ii) Swimming: MNRs capable of swimming can be used for tasks such as clearing clogged arteries or delivering therapeutic agents to specific organs or tissues. Their swimming motion allows them to propel through fluids, facilitating navigation within the body. Some special swimming pattern like spiral render them ability to navigate complex environments such as vascular networks and tumor microenvironments. iii) Crawling: MNRs with crawling capabilities can be employed for tasks like tissue exploration or wound healing. Their crawling motion enables them to navigate through intricate structures, such as the digestive tract or damaged tissue, for diagnostic or therapeutic purposes.

## 5 Applications of electrically controlled MNRs

### 5.1 Overall application scenarios of electrically controlled MNRs

Electrically controlled MNRs have made significant advancements in design and fabrication. Compared to other manipulation methods, the major advantage of electrically controlled MNRs is their low cost and non-contact manipulation capability (Wang et al., 2021). Although magnetically controlled MNRs possess similar properties, they fall slightly behind in terms of manipulation ability, precision, and real-time responsiveness (Han et al., 2018; Wang et al., 2021). MNRs with conductive or semiconductive properties can be precisely controlled and exhibit multidirectional movement or rotation in three-dimensional space through changes in external electric fields (Fan et al., 2005; Guo et al., 2018; Han et al., 2018). Kim et al. (2014) designed an ordered array of nanoscale motors through bottom-up assembly, where nanowires act as rotors, patterned nanomagnets serve as bearings, and quadrupole microelectrodes function as stators. Under the influence of an electric field, the nanomotor array can perform chiral rotations with controllable angles and speeds (exceeding 18,000 r.p.m.). This research demonstrates the significant implications for nanoelectromechanical systems, nanomedicine, microfluidics, and on-chip laboratory architectures. However, the application of electrically controlled MNRs is still limited in the human body, especially in complex environments such as the bloodstream, due to the rapid decay of electric fields with distance and their instability in high-particle environments (Wang and Gao, 2012). Currently, mainstream research suggests that electrically controlled MNRs have a unique advantage in size control. Due to dielectrophoretic forces, electric fields can achieve precise control at the microscale (Velev et al., 2003; Han et al., 2018), whereas the size of magnetically controlled MNRs is typically limited by magnetic materials (Gao Y. et al., 2021). It can be expected that, once the challenges of interference resistance and sustained operation of electrically controlled MNRs are addressed, they will demonstrate promising performance in targeted tumor therapy, drug delivery, nucleic acid biomarker detection, diagnostics, sensing, microsurgery, blood clot ablation, and wound healing.

**FIGURE 4:**
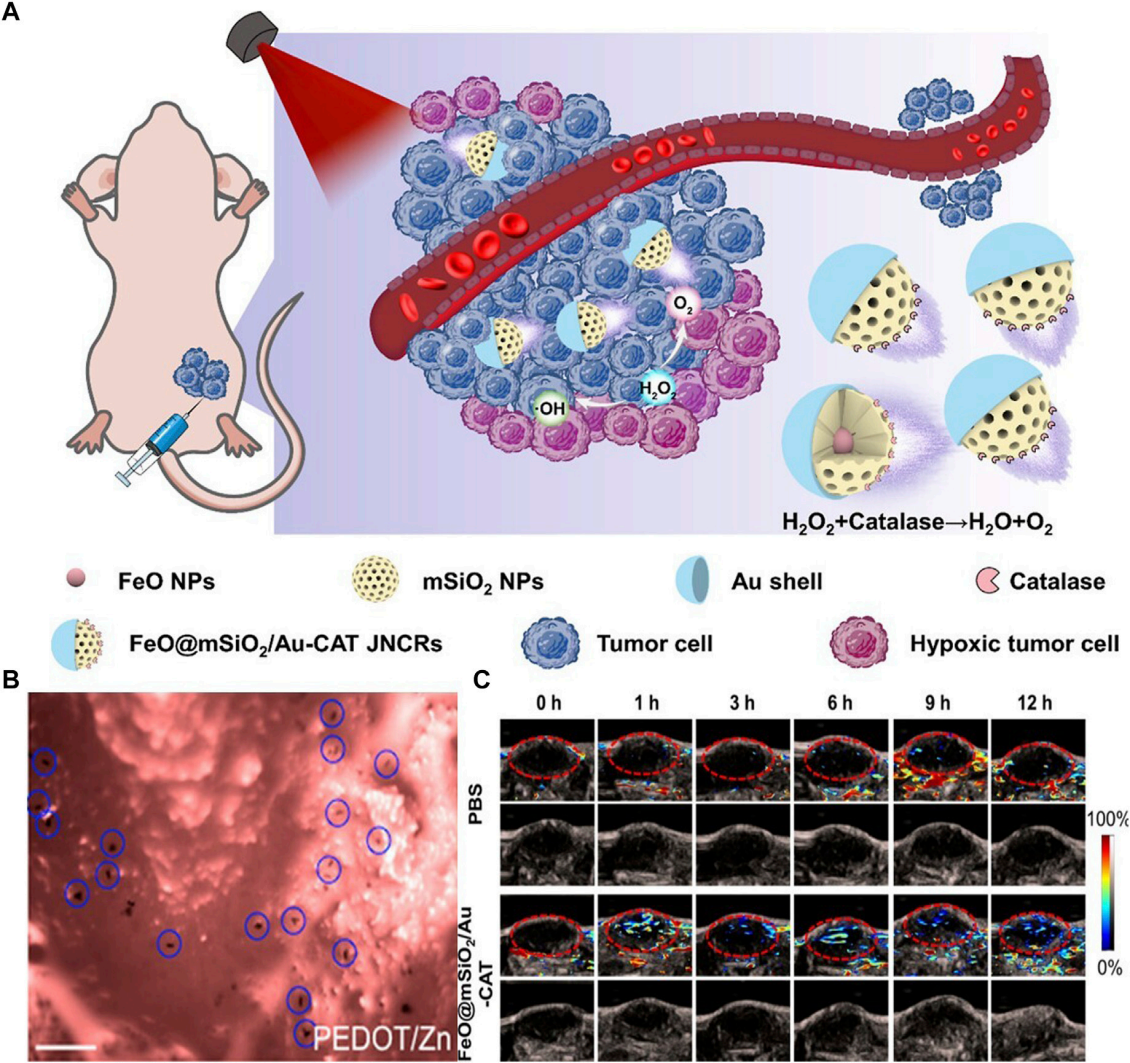
**(A)** Mechanisms of Self-Propelled Janus Nanocatalytic Robots for 4T1 tumor-bearing mice (Sun et al., 2023). 2023 American Chemical Society **(B)** Retention of PEDOT/Zn micromotors on mouse gastric mucosal tissues. Scale bars, 100 μm (Gao et al., 2015b). 2014 American Chemical Society **(C)** Saturated oxygen (sO2) levels in the tumour vasculature after saline or Janus nanocatalytic robots injections at 0, 1, 3, 6, 9 and 12 h (Sun et al., 2023). 2023 American Chemical Society.

On the other hand, research on MNRs propelled by electrochemical reactions has reached relatively mature stages. The electrochemical reactions occurring in asymmetric particles provide a strong theoretical foundation for self-propelled electrically controlled MNRs (Paxton et al., 2004; Paxton et al., 2005; Wang et al., 2013). The electrochemical and fluidic properties resulting from redox reactions (Luo et al., 2019) enable MNRs with electrochemical capabilities to exhibit good performance in self-propulsion and sustained propulsion (Paxton et al., 2005; Calvo-Marzal et al., 2010; Kümmel et al., 2013; Dai et al., 2016). Sun et al. (2023) combined MNRs with nano-catalytic medicine to achieve deep tumor penetration induced by self-propulsion and catalytic reaction-triggered tumor therapy *in vivo* (Figures 4A, C). They utilized self-propelled Janus nano-catalytic robots (JNCR) guided by magnetic resonance imaging (MRI) for enhanced tumor treatment inside the body. JNCR exhibited active mobility in the H2O2 solution. Compared to passive nanoparticles, these self-propelled JNCRs could penetrate deeper into tumors after intratumoral injection, thereby improving tumor treatment efficacy. These robots demonstrated biocompatibility in mouse models and hold the potential for integrating MNRs with nano-catalytic medicine, thereby improving tumor therapy and achieving clinical translation. However, H2O2 exhibits certain biological toxicity in the body (Wang et al., 2021), and further exploration is needed for the *in vivo* application of related electro-catalytic-driven microelectric motors. Conversely, non-contact electric fields have a greater advantage in terms of safety. Chen et al. (2014) proposed a wireless-powered microrobot based on an Archimedean spiral, which possesses a highly integrated active motion module. The researchers fabricated and tested a prototype for wireless capsule endoscopy (WCE) using radiofrequency-based power transfer. Through design modifications, a cylindrical ferrite core was added to the receiving coil array, significantly improving the coupling efficiency (up to 12%). Such designs can also achieve functionalities such as targeted propulsion and integration with imaging techniques, while significantly enhancing safety.

### 5.2 Applications of Janus particles in electrically controlled MNRs

Janus particles with distinct dielectric properties have emerged as a highly promising choice for electrically controlled MNRs. These particles possess induced charges and an asymmetric distribution of electric dipole s (Demirörs et al., 2018), enabling their propulsion and manipulation through ICEP (Lee et al., 2023), Lee et al. (2023) discovered that magnetic locking assemblies of metallic-dielectric Janus particles exhibit five distinct motion states under the influence of ICEP, with four of them achievable using Janus dimers alone. Furthermore, the asymmetric nature of Janus particles leads to an imbalanced electrokinetic flow on both sides of the particle (Ma et al., 2015), endowing them with self-propulsion capabilities. With this combination of characteristics, Janus particles have demonstrated significant potential in biomedical applications such as drug delivery, cell manipulation, and targeted transport. In all these applications, precise positioning and navigation of Janus particles are often crucial initial steps.

Current mainstream MNRs employ various methods for localization and guidance, including magnetic (Diller et al., 2013; Peyer et al., 2013; Servant et al., 2015; Li et al., 2019; Xie et al., 2019; Fang et al., 2023), optical/thermal (Wang J. et al., 2019; Xie et al., 2019; Dai et al., 2022; Wang et al., 2023), and acoustic (Abbas et al., 2020; Das et al., 2022; Wu et al., 2023) methods, as well as physical constraints through microstructure fabrication and microfluidic manipulation (Katuri et al., 2016; Patteson et al., 2016). However, these approaches are often limited by the physical structures of the materials themselves or the complex interactions between materials and microfluidic channels, making it challenging to explore new directions and optimize their performance. Despite these limitations, the development of electrically controlled Janus particles, particularly those controlled by ICEP, has been relatively limited until the introduction of a self-powered microelectromotor by Yoshizumi et al. (2015). This microelectromotor utilizes alternating-current electroosmosis (ACEO) and positive dielectrophoresis (pDEP) for control and is capable of simple, low-cost two-dimensional motion. The micro electromotor is composed of a gold/platinum bilayer Janus particle and can be individually controlled for self-propulsion using grid electrodes formed at the bottom and top of microchannels, eliminating the need for external guiding devices such as multi-axis manipulators. The behavior of 5 μm PS/Pt/Au microelectromotors was investigated in deionized water and 1M H2O2 solution. Experimental results revealed that the MNRs possessed a negative zeta potential in the water solution. When immersed in a fuel solution, the electrochemical reactions of gold and platinum induced an electric field resulting from charge separation, causing electrophoretic fluid to flow along the surface of the microelectromotor towards the negative electrode, thereby enabling its motion. However, similar to the design by Sun et al., the self-propelled motion of the Janus particle microelectromotor was observed only in the presence of H2O2 solution, with an average velocity of approximately 7 μm/s. Additionally, Clausius-Mossotti factor (CMF) measurements conducted on PS, PS/Pt, and PS/Pt/Au particles revealed that PS particles exhibited behavior similar to negative dielectrophoresis at higher frequencies, while PS/Pt and PS/Pt/Au particles exhibited negative dielectrophoresis-like behavior at all frequencies, attributed to enhanced polarization from the metal shell structure. At low frequencies, PS, PS/Pt, and PS/Pt/Au particles were dragged toward the center of the ITO electrode due to ACEO effects, and pDEP effectively attracted the MNRs to the edge of the electrode. Such structural and design considerations provide novel ideas for the two-dimensional motion of electrically controlled Janus particle microelectromotors. Building upon this research, Zhang et al. (2019) proposed a similar collection and anchoring mechanism for Janus particles under the propulsion of ICEP. The team reported a universal strategy for propelling, constraining, and collecting metal-dielectric Janus microspheres using an interdigitated microelectrode design. Interdigitated microelectrodes exhibit rich alternating-current electrodynamic characteristics and are widely employed in applications such as the detection and separation of biological samples, as well as biochemical and electrochemical sensing. It was observed that particles could be propelled along the center of the electrode towards both ends via ICEP and subsequently trapped by electroosmosis, without the need for additional fuel. Furthermore, the particles rotated at the closed end of the electrode, decelerated, and accumulated at the open end. By simply modifying the electrode configuration, a maze pattern composed of serpentine chains could be formed. This study presents a versatile electrode-based strategy for controlling one-dimensional transport and aggregation of MNRs in a configurable, switchable, and non-contact manner, offering potential benefits for fundamental research and applications such as sensing, sorting, and transport.

Compared to static positioning, the control of Janus particles’ motion holds greater clinical value. Lee et al. (2019) discovered that asymmetric metallic patches on the surface of spherical colloids can be driven by an alternating electric field, guiding the colloids to move along nonlinear helical trajectories. By altering the size and shape of the patches, the details of particle motion, such as trajectory speed and radius, can be adjusted. Particles with approximately mirror-symmetric triangular patches can perform helical motion rather than linear propulsion. In porous membranes, helical trajectories offer functional advantages for particle navigation compared to linear propulsion. This implies that Janus particles propelled in a helical manner are an ideal modality in complex environments such as the human body. Moreover, as particle symmetry is further reduced, the motion patterns become increasingly complex. By comparing particles with complex helical trajectory motion to particles with ordinary linear motion, the research team found that particles with complex trajectories are more likely to find and traverse channels in porous matrices, further demonstrating the advantages of helical motion for *in vivo* applications. Similarly, Demirörs et al. (2021) found that Janus particles are influenced by external electric fields and the chemical properties of their contact surfaces, allowing the particles to be coupled to rolling motion in orthogonal electric fields under certain conditions, and estimating the contact surface friction properties using derived mathematical ratios. Rolling motion mimics migration behaviors observed in leukocytes and macrophages, indicating that electrically controlled Janus particles’ motion is not limited to pure fluidic environments but can also occur at the fluid-substrate interface. Furthermore, the rolling response of Janus microrobots can be utilized for reversible cargo capture and release using electric fields, enabling the manipulation of cargo particles at the microscale. By combining functional molecules, Janus microrobots can be used to create microscale chemical gradients or achieve programmable release with spatiotemporal control, applying to bioanalytical platforms for drug screening, medical diagnostics, or fundamental biological research.

Another key feature of Janus particles is their self-propulsion ability. Compared to external field manipulation, self-propelled Janus particles offer the following advantages: i) better mobility in complex environments such as high ionic environments like blood, ii) spontaneous emergence of collective behaviors, and iii) potential for self-assembly. This is due to the generation of surface forces upon breaking symmetry, resulting in a phenomenon known as self-electrophoresis (Boymelgreen et al., 2016) Similar concepts have been demonstrated in early zinc-based micromotors. Gao et al. (2015a) conducted *in vivo* studies of artificial micromotors in mice through oral administration (Figure 4B). The results showed that propulsion forces driven by gastric acid could effectively enhance the binding and retention of micromotors and their payloads on the gastric wall. The micromotor body gradually dissolves in gastric acid and autonomously releases the carried payload without leaving any toxic residues. In addition to drug delivery, self-propelled micromotors also assist in detoxification (Orozco et al., 2013) and self-healing systems (Li et al., 2015). Building upon such research, asymmetric self-propelled Janus particles with more complex structures naturally hold greater development potential. Das and Yossifon, (2023)employed optoelectric tweezers to achieve simultaneous manipulation of Janus active particles and electrically driven self-propulsion while discretizing the external field space. Microscopic objects were manipulated using optically induced dielectrophoresis, generating and controlling multiple beams of light through a digital micromirror device (DMD) in a dynamic and programmable manner. Additionally, by controlling the number and types of illuminated regions, the self-assembly of active particles into specific configurations can be guided. Compared to random assembly, this system allows for deterministic combination of non-active particles, forming complex hybrid structures. The study demonstrates that sufficiently large active particles can uniformly manipulate synthetic/biological cargo particles. Moreover, the photoelectric effects generated by manipulating particles can also induce effects like electroporation on cells, indicating the potential realization of gene transfection, cell fusion, and cell separation for individual target cells using this approach. Similarly, Peng et al. (2020), inspired by the motion patterns of *Escherichia coli*, developed a photothermal-electrically driven Janus particle that exhibits rotation within a plane under a temperature gradient-induced electric field, in addition to conventional photoelectric effects. By adjusting the rotation laser beam’s timing, the particle can be positioned in any desired direction, enabling efficient active control of the swimming direction. Through dark-field optical imaging and feedback control algorithms, autonomous propulsion and navigation of MNRs have been achieved. This approach overcomes the disorder in long-timescale motion direction caused by random Brownian motion of Janus particles.

As research progresses, an increasing number of scientists recognize the limitations of single-driven MNRs, especially Janus particles, in complex application environments due to strict energy and environmental constraints (Xu et al., 2017). MNRs with different responses often require different asymmetric materials, such as magnetic Janus particles composed of Fe2O3/ZnFe2O4/Mn2O3 (Yang et al., 2023), asymmetrically sound-pressure-driven Au nanowires (Wang et al., 2012), and electrochemically catalyzed Pt/Au microparticles (Paxton et al., 2004). Zheng et al. [116] developed a double-layered bowl-shaped structure consisting of platinum and α-Fe2O3, integrating five driving engines: light, sound, magnetic, electric, and chemical. Under an alternating electric field, the inner platinum and outer α-Fe2O3 layers exhibit asymmetric polarization, inducing motion through charge-induced electroosmosis (ICEO). The other four propulsion mechanisms enhance the performance of the micro-robot in specific applications. These multi-driven Janus particles may possess unique adaptability and advantages in complex biological environments.

Overall, Janus particles with asymmetric chemical structures are excellent materials for electric field-driven and self-propelled systems, offering the following advantages: i) Multifunctionality: The two different surfaces of Janus particles can exhibit distinct functionalities. For example, one side may possess biocompatibility for drug binding or act as a biosensor, while the other side may have conductivity for power generation or catalytic activity. In future developments, these surfaces could be interchangeable to adapt to different biological environments such as blood, blood vessel walls, and tissue structures. ii) Controllability: Under certain conditions, such as various external fields and electron tweezers microfluidic chip control, Janus particles can be precisely positioned and manipulated within a certain range. The influence of external field-driven and self-propelled Janus particles has significant implications for applications such as targeted tumor therapy and precise cell manipulation. iii) Aggregability: Janus particles can be precisely positioned and aggregated under certain electrical field stimulations, such as discrete electric fields and optoelectric chips, leading to cluster effects and unexpected functionalities and motion patterns. This offers limitless possibilities and surprises in the future development of biomedical applications.

However, Janus particles also face some challenges: i) Difficulty in preparation: The synthesis of Janus particles often requires special methods and conditions, which may increase complexity and cost. ii) Biocompatibility: Some Janus particles require biologically harmful environments such as H2O2, and most of the research on Janus particles is limited to *in vitro* studies, lacking validation for biocompatibility. iii) Stability: Janus particles composed of inert materials like Au/Pt exhibit relatively high stability, but the stability and durability of other active materials such as iron and zinc need further consideration.

### 5.3 Electrophysiological processes in bionic electro-controlled MNRs

To address the issues of large size, complex manufacturing, low efficiency, high frictional losses, and generation of hazardous waste associated with traditional rigid MNRs (Wang, 2009; Alizadeh et al., 2020; Soto et al., 2020; Huang et al., 2022), in addition to artificial micro/nanoparticles, bionic micro motors have also rapidly developed in the past decade. Bionic micromotors can be broadly categorized into three types: MNRs that mimic/utilize bacteria/algae (Ma et al., 2012; Park et al., 2013; Rogowski et al., 2020; Rogowski et al., 2021; Xie et al., 2021; Gwisai et al., 2022), those that mimic/utilize sperm (Magdanz et al., 2013; Medina-Sánchez et al., 2016; Chen et al., 2021), and bionic micro motors that mimic muscles. Shi and Yeatman (2021) defined a class of responsive materials or devices that are small in scale, low in stiffness, and capable of replicating the core functionality of natural muscles as artificial muscles. These artificial muscles exhibit various modes of response, including electrical (Jang et al., 2021), pressure-based (Jang et al., 2015; Jang et al., 2017) chemical environment-based (Mashayekhi Mazar et al., 2019), thermal (Haines et al., 2014; Mirvakili and Hunter, 2017), and magnetic (Lee et al., 2018) responses. In this review, electrically stimulated artificial muscles are considered as part of electrically controlled MNRs.

**FIGURE 5:**
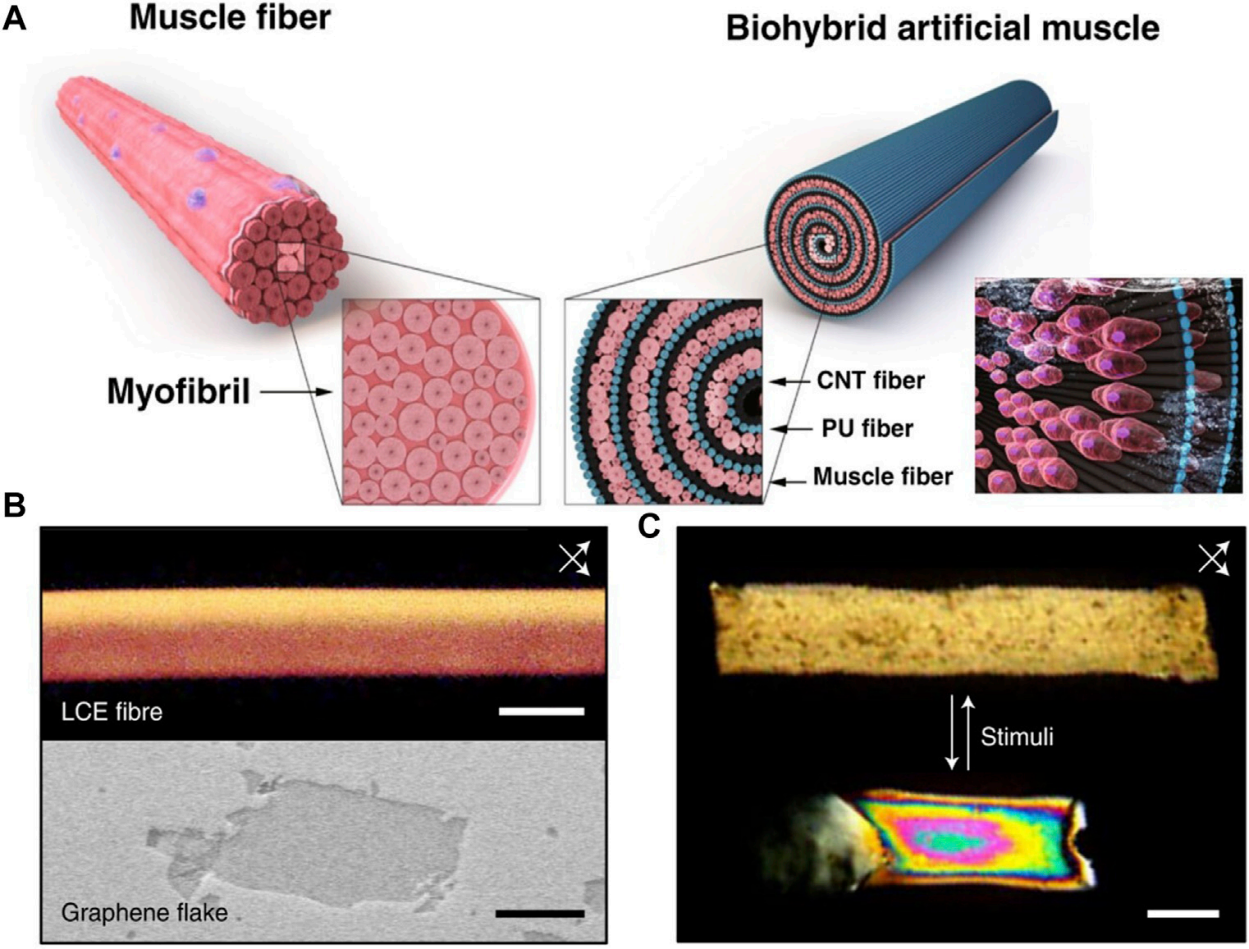
**(A)** The structural similarities and differences between natural muscles and artificial muscles (Jang et al., 2021) 2021, The Author (s). **(B)** Polarized Optical Microscope of Liquid Crystal Formation observed at 45° angles with respect to cross-polarizers (top) and Scanning Electron Microscope image of an Electrographite flake (bottom) (Kim et al., 2022). 2022, The Author (s). **(C)** The shape deformation of artificial muscles during relaxation and contraction (Kim et al., 2022) 2022, The Author (s).

Electrically stimulated artificial muscles often utilize stimulation signals directly to achieve contraction. Jang et al. (2021), inspired by skeletal muscle myofibrils, developed a bundle-shaped biohybrid artificial muscle (Figure 5A). By integrating skeletal muscle cells with hydrophilic polyurethane and carbon nanotube nanofibers, they created a structure resembling natural skeletal muscle fibers. The nanofibers provided a stretchable scaffold, similar to the framework of actin and myosin, while the incorporation of skeletal muscle fibers helped drive the biohybrid artificial muscle. Reversible contraction of the biohybrid artificial muscle can be achieved through electric field stimulation. This electrically driven artificial muscle holds tremendous potential for driving motion and drug delivery systems in implantable medical robots. Furthermore, the integration of biohybrid artificial muscles controlled by motor neurons is of significant importance for brain-machine interfaces.

In addition to bionic muscles prepared through biohybrid approaches, ion polymer actuators made of electrically lightweight, low-power, active polymers have also received attention (Shahinpoor and Kim, 2005; Khan et al., 2015). These actuators are flexible, lightweight, low-power, and can be processed at the nanoscale, making them ideal for artificial muscles. Khan et al. (2019) synthesized ion-exchange polymer films based on polypyrrole/polyvinyl alcohol (PPy/PVA), incorporating PEDOT:PSS/SWNT/IL electrodes that can change conformation through externally applied voltage. To investigate size-dependent electromechanical actuation performance, the researchers fabricated ion polymer actuator films with four different sizes of PPy nanoparticles. Compared to other expensive perfluorinated polymer-based actuators, the PPy/PVA polymer composite films showed enhanced electrical performance and tip deflection performance, with the electrodes utilizing a hybrid PEDOT:PSS/SWNT/IL electrode film. Based on this, a microgripper device with two fingers composed of PPy/PVA/EL ion-exchange polymer films was developed. MNRs designed with this approach exhibit electrophysiological functionality similar to natural muscles, and their ability to change conformation through externally applied voltage provides additional functional potential beyond natural muscles. In the field of implantable medical robots, such bionic muscles can meet macro-scale requirements with a micro-scale configuration, enabling tasks such as object grasping and precision surgery.

Building upon artificial muscles, researchers believe that the piezoelectric properties of artificial muscles can be effectively extended to artificial cochlea. Jang et al. (2015) developed a piezoelectric artificial basilar membrane (ABM) composed of a microelectromechanical system (MEMS) cantilever array, which emulates the tonotopic characteristics of the cochlea and produces clear tones within the audible frequency range. Through experiments conducted on animal models, the characteristics of ABM as a potential front-end for cochlear implant applications were validated. The frequency selectivity of ABM was confirmed by measuring electrically evoked auditory brainstem responses (EABR).

Overall, the advantages of bionic artificial muscles are evident. As an ideal microactuator mechanism, they possess the following features: i) Soft and resilient mechanical characteristics: Artificial muscles exhibit flexibility and elasticity similar to natural muscles, enabling highly deformable motions (Figures 5B, C). ii) Flexible driving mechanisms: Microactuators provide precise control and adjustment, allowing fine movements and power output of artificial muscles. iii) Small size and high efficiency: Microactuator-driven artificial muscles demonstrate acceptable efficiency at the micrometer to sub-centimeter scale, making them highly suitable for microsystem applications. This potential opens up avenues for development in the following areas: i) Robotics and human-machine interaction: Microactuator-driven artificial muscles can be employed in the motion and operation of robots, enabling more natural and flexible human-machine interaction. ii) Prosthetics and rehabilitation devices: Artificial muscles can be utilized in the production of lightweight and efficient prosthetics and rehabilitation devices, offering improved movement and functional recovery. iii) Medical instruments: Microactuator-driven artificial muscles can be employed in microscale medical instruments such as endoscopes and minimally invasive surgical devices, enabling precise manipulation and control. iv) Flexible electronic products: Artificial muscles can be integrated into flexible electronic products, such as wearable devices and smart textiles, for a more comfortable and adaptive user experience. v) Biomedical research: Artificial muscles can be utilized in biomedical research, including biomimetic models and tissue engineering, aiding in the understanding and simulation of biological system movements and functions.

## 6 Discussion

In conclusion, although the clinical application of electrically controlled MNRs is still in its early stages, their complex motion patterns, self-propulsion capabilities, and deformability have shown promising potential. However, the practicality and stability of electrically controlled MNRs are still subject to exploration due to fabrication challenges and the highly ionic environment within the human body.

In the future, qualified electrically controlled MNRs should possess the following characteristics: i) Good biocompatibility to avoid recognition as foreign objects and clearance by the immune system; ii) Dual control of external fields and self-propulsion to accomplish tasks in complex environments; iii) Strong deformability allowing a single robot to perform multiple tasks; iv) Low-cost fabrication with streamlined production.

As research progresses, the applications of electrically controlled MNRs will become increasingly diverse and suitable for various purposes. With the help of the aforementioned characteristics, electrically controlled MNRs hold tremendous potential for applications in the following areas: i) Targeted drug delivery: This form of targeted delivery based on electrically controlled MNRs can enhance the efficacy of drugs, reduce side effects, and enable more effective treatment methods. ii) Diagnostic monitoring of biomarkers and pathological changes: By performing monitoring within the body, these MNRs can provide more accurate, sensitive, and real-time diagnostic results. iii) Minimally invasive surgery and interventional therapy: Electrically controlled MNRs can be utilized for minimally invasive surgical procedures and interventional therapies, thereby reducing surgical trauma and recovery time. iv) Cancer treatment: In the fields of early cancer detection, targeted drug delivery to tumors, and precise radiotherapy, electrically controlled MNRs can improve treatment efficacy and reduce side effects.
